# Measuring health-related quality of life in chronic headache: A comparative evaluation of the Chronic Headache Quality of Life Questionnaire and Headache Impact Test (HIT-6)

**DOI:** 10.1177/03331024211006045

**Published:** 2021-05-04

**Authors:** Kirstie L Haywood, Felix Achana, Vivien Nichols, Gemma Pearce, Barbara Box, Lynne Muldoon, Shilpa Patel, Frances Griffiths, Kimberly Stewart, Martin Underwood, Manjit M Matharu, on behalf of the CHESS Team

**Affiliations:** 1 Warwick Research in Nursing, Warwick Medical School, University of Warwick, Coventry, UK; 2 Warwick Clinical Trials Unit, Warwick Medical School, University of Warwick, Coventry, UK; 3 Nuffield Department of Primary Care Health Sciences, Oxford University, Oxford, UK; 4 School of Psychology, Social and Behavioural Sciences, Coventry University, Coventry, UK; 5 Social Science and Systems in Health, Warwick Medical School, University of Warwick, Coventry, UK; 6 University Hospitals Coventry and Warwickshire, Coventry, UK; 7 The Headache Group, National Hospital for Neurology and Neurosurgery, University College of London Hospitals NHS Foundation Trust, AC1 London, UK

**Keywords:** Headache Impact Test, psychometric evaluation, chronic headache, quality of life, outcome measures

## Abstract

**Objective:**

To compare the quality and acceptability of a new headache-specific patient-reported measure, the Chronic Headache Quality of Life Questionnaire (CHQLQ) with the six-item Headache Impact Test (HIT-6), in people meeting an epidemiological definition of chronic headaches.

**Methods:**

Participants in the feasibility stage of the Chronic Headache Education and Self-management Study (CHESS) (n = 130) completed measures three times during a 12-week prospective cohort study. Data quality, measurement acceptability, reliability, validity, responsiveness to change, and score interpretation were determined. Semi-structured cognitive interviews explored measurement relevance, acceptability, clarity, and comprehensiveness.

**Results:**

Both measures were well completed with few missing items. The CHQLQ’s inclusion of emotional wellbeing items increased its relevance to participant’s experience of chronic headache. End effects were present at item level only for both measures. Structural assessment supported the three and one-factor solutions of the CHQLQ and HIT-6, respectively. Both the CHQLQ (range 0.87 to 0.94) and HIT-6 (0.90) were internally consistent, with acceptable temporal stability over 2 weeks (CHQLQ range 0.74 to 0.80; HIT-6 0.86). Both measures responded to change in headache-specific health at 12 weeks (CHQLQ smallest detectable change (improvement) range 3 to 5; HIT-6 2.1).

**Conclusions:**

While both measures are structurally valid, internally consistent, temporally stable, and responsive to change, the CHQLQ has greater relevance to the patient experience of chronic headache.

**Trial registration number:** ISRCTN79708100. Registered 16th December 2015, http://www.isrctn.com/ISRCTN79708100

## Introduction

Chronic headaches, which can be defined epidemiologically as headaches on 15 or more days per month for at least 3 months (1–3), have profound effects on people’s lives. Those affected describe strained relationships, and that the spectre of headaches can be a crucial driver of their behaviour (4). When testing treatments for these chronic headache disorders, an international, multi-stakeholder consensus process rated the measurement of the overall health impact of chronic headaches as being at least as important as counting headache days (5). These health impacts should be assessed using patient-reported outcome measures (PROMs) with robust evidence of measurement quality, relevance, and acceptability (5,6). There is substantial heterogeneity in PROMS used in trials of headache disorders (7).

A 2018 systematic review of PROMS for headaches found the strongest, albeit limited, evidence was for two headache-specific measures (7), the Migraine-Specific Questionnaire (MSQ v2.1) (8) and the six-item Headache Impact Test (HIT-6) (9). However, essential evidence of data quality and interpretation, reliability, and responsiveness was mostly absent or of insufficient quality. Moreover, the relevance and acceptability of these measures to people with headache were not explored. The use of PROMs that lack relevance to patients, and hence fail to capture the outcomes that matter, places an unnecessary burden on patients, and maybe judged to be unethical (10).

We report here on a mixed-methods comparative evaluation of the measurement and practical properties of the HIT-6 and an adaption of the MSQ v2.1 to make it suitable for people with unspecified chronic headache disorders – the Chronic Headache Quality of Life Questionnaire (CHQLQv1.0).

## Methods

The Chronic Headache Education and Self-Management Study (CHESS) is a programme grant funded by the UK’s National Institute for Health Research (RP-PG-1212-20018) to test the effectiveness of a supportive self-management intervention for people living with chronic headache disorders (11). This current work forms part of the feasibility study, reported elsewhere (January 2016 to April 2017) (Black Country Research Ethics Committee (15/WM/0165)) (12). In summary, participants completed questionnaires on three occasions during a 12-week prospective cohort study (baseline, 2 and 12 weeks).

### Study population

We recruited people living with chronic headaches, predominantly chronic migraine or chronic tension-type headache, from general practices in the West Midlands region of the UK. Practices wrote to people who had, in the previous 2 years, consulted for headaches or had a prescription for a migraine-specific drug (i.e. triptans/pizotifen), inviting expression of interest in the study. In a subsequent telephone interview, study team members assessed if participants met an epidemiological definition of chronic headaches: Headache for 15 or more days per month for at least 3 months (1–3). For this validation of a generic headache-related quality of life outcome that is not diagnosis specific, this is the appropriate population. However, as part of this overall programme of work, we also validated a classification interview in this population. Of the 131 people included in this report, 107 (82%) also had paired telephone interviews with research nurses and doctors from the National Migraine Centre. The final classification was: Definite chronic migraine (59; 55%), probable chronic migraine (40; 37%) chronic tension-type headache (6; 6%), cluster headache (2; 2%), hemicrania continua (1; 1%). Over half, 44/74 (59%), also had medication overuse defined as “headache occurring on 15 or more days per month taking acute or symptomatic headache medication (on 10/15 or more days per month, depending on the medication) for more than 3 months”. The sample size was driven by requirements for validation of a chronic headache classification interview. This work is described in detail elsewhere (13).

### Patient-reported outcome measures

The feasibility study included general headache-specific (not diagnosis-specific), generic and domain-specific measures and a headache-specific health transition question (detailed in Appendix 1). The CHQLQ is a 14-item questionnaire, which assesses the functional aspects of headache-related quality of life, producing three domain scores (role prevention, role restriction, and emotional function) (8). Modification of the CHQLQ from the MSQ (v2.1) simply involved replacing the word ‘migraines’ with ‘headaches’ throughout the questionnaire. The HIT-6 is a 6-item questionnaire, which produces a single index score of headache impact on functional ability (9). Participants self-completed postal questionnaires at baseline, 2 and 12 weeks.

### Analysis

Psychometric properties of the measures were compared ((14,15); Appendix 2).

#### Data quality and interpretability

Item-scale characteristics, completion rates (missing data) and percentage of computable scale scores are reported (15,16). Interpretability was informed by evidence of end effects and calculation of the minimal important change (MIC) – the smallest change in score perceived as important by participants) (15) – calculated as the mean change score for people reporting “minimal change” in their headache at 12 weeks.

#### Structural validity and internal consistency

An exploratory factor analysis on baseline data hypothesised that the CHQLQ’s original three-factor solution would be retained. Absolute item loadings ≥0.45 were accepted as sufficient correlation with a principal component to support domain inclusion. Confirmatory factor analysis was then used to confirm the three- and one-factor structures of the CHQLQ and HIT-6, respectively. Factor loadings exceeding 0.3–0.4 were judged to be meaningful (15–17). Internal consistency was assessed with Cronbach’s alpha (15,16) values between 0.7 and 0.90 suggest a good to excellent agreement between items and the total (domain) score (15,16).

#### Reliability and measurement error

Two-week test-retest reliability (intra-class correlation coefficient (ICC 2,1)) was assessed in those indicating no change in their headache. We calculated the standard error of measurement (SEM) to determine the extent of absolute measurement error (6,18,19). The SEM supports score interpretation by accounting for variability, or error, in measurement – only a change greater than measurement error is considered ‘real’ (18). The SEM was subsequently converted into the smallest detectable change (SDC), representing the smallest change in score that is greater than measurement error; the SDC was calculated for individuals and for groups (19,20). The SDC allows one to rule out measurement error (i.e. distinguishing measurement error from true change) when assessing the reliability of a self-reported measure to detect change in health status. Thus, a score change greater than the SDC value is necessary to provide evidence of true change (improvement or deterioration) in health-status.

#### Construct validity

Score correlation between measures was assessed to evaluate convergent validity (Pearson’s correlation coefficient). Hypothesised theoretical associations were considered *a priori* (Appendix 2).

#### Responsiveness

Responsiveness reflects the ability of a measure to detect real change in health that is greater than measurement error.

- *Smallest detectable change (SDC)*

We calculated the absolute measurement error at 12 weeks (standard error of measurement (SEM) and the smallest detectable change (SDC)), to represent the smallest change in score that is greater than measurement error in patients reporting change in headache at 12 weeks. We calculated the minimal important change (MIC) as the mean change in those reporting minimal improvement or deterioration at 12 weeks. We calculated the minimal important clinical difference (MICD) as the mean change in score in those who are “somewhat better” minus the mean change in those who are the same at 12 weeks (6,16).

(ii) *Criterion-based assessment*

Receiver operating characteristic (ROC) curves were calculated to assess the ability of measures to discriminate between people whose headache had improved or deteriorated (on headache-specific transition question) at 12 weeks (16). An area under the curve (AUC) score of > 0.70 is considered sufficiently discriminatory; an AUC of 0.5 suggests no discriminatory power.

(iii) *Effect size (ES) and standardized response mean (SRM)*

The ES and SRM were calculated for subgroups of patients in each health transition category. The main hypotheses we tested were: ES and SRM would be <0.2 for patients who reported no change in headache; >0.2 for patients reporting a slight improvement; >0.5 for patients reporting improvement (much better); greater for patients indicating an improvement in their headache than those indicating no change.

### Content validity

Semi-structured cognitive interviews were conducted within 24 h of questionnaire self-completion with a purposive sample (age, gender, headache type) of participants. Measurement relevance, acceptability, clarity, and comprehensiveness were explored (21,22). Overarching questions explored how patients determined headache improvement, and if specific questions were missing. Interviews continued until thematic saturation was achieved; they were audio-recorded, transcribed verbatim, and checked for accuracy (VN). We used framework analysis (23) and cross-case comparison to generate themes. NVivo software (QSR International Pty Ltd. Version 11, 2015) supported data organisation. Data were independently explored by two researchers (VN, KH); emergent themes were discussed and interpreted with a third researcher (FG) and with two of our patient research partners (BB, LM).

## Results

We recruited 131 people: 130, 115 (88%) and 103 (79%) questionnaires were completed at baseline, 2 and 12 weeks, respectively (Table 1) (11).

**Table 1.** Patient characteristics at baseline and follow-up.

	Baseline (n = 130)	2-week response (n = 115)		12-week response (n = 103)	
Characteristic	n (%)	n (%)	p-value^1^	n (%)	p-value^1^
Age (years)					
Mean (SD)	48.7 (13.2)	49.8 (13.1)	0.006	49.8 (13.1)	0.006
Range	21–77	21–77		21–77	
Gender					0.447
Female	107 (82.3%)	93 (81%)	0.483	84 (82%)	
Unknown	2 (1.5%)	2 (2%)	–	1 (1%)	
Ethnicity					0.004
White	124 (95.4%)	112 (97%)	0.002	101 (98%)	
Non-white	5 (3.8%)	2 (2%)	0.002	1 (1%)	
Not reported	1 (0.8%)	1 (1%)	–	1 (1%)	
Left school at					0.46
Age 13–16	35 (26.9%)	33 (29%)	0.085	29 (28%)	
Age 17–19	47 (36.2%)	41 (36%)	0.085	37 (36%)	
Age 20 or over	43 (33.1%)	37 (32%)	0.085	34 (33%)	
In full-time education	3 (2.3%)	3 (3%)		2 (2%)	
Other	1 (0.8%)	0	–	–	
Not reported	1 (0.8%)	1 (1%)	–	1(1%)	
Employment status					0.487
Employed	85 (65.4%)	73 (63%)	0.724	65 (63%)	
Retired from paid work	22 (16.9%)	21 (18%)	0.724	20 (19%)	
At school or in full time education	2 (1.5%)	2 (2%)	–	2 (2%)	
Looking after your home/family	11 (8.5%)	9 (8%)	0.724	8 (8%)	
Unable to work due to long term sickness	3 (2.3%)	3 (3%)	–	3 (3%)	
Other	2 (1.5%)	2 (2%)	–	2 (2%)	
Not reported	5 (3.8%)	5 (4%)	–	3 (3%)	
Type of headache					
Definite chronic migraine	59 (45.4%)	57 (50%)	<0.001	48 (47%)	<0.001
Probable chronic migraine	40 (30.8%)	37 (32%)		37 (36%)	
Chronic tension	6 (4.6%)	5 (4%)		6 (6%)	
Unknown	25 (19.2%)	16 (14%)		12 (12%)	
Medication overuse					
Yes	68 (52.3%)	66 (57%)	<0.001	57 (55%)	<0.001
No	37 (28.5%)	33 (29%)		34 (33%)	
Unknown	25 (19.2%)	16 (14%)		12 (12%)	

^1^p-values compare baseline characteristics of responders and non-responders at the 2-week and 12-week follow-up assessment point.

### Data quality and interpretability

Item missing data for the CHQLQ was low (range 0% to 3%); domain scores were computable for 96% (role prevention), 97% (role restriction) and 100% (emotional function) of respondents (Table 2). All response options were endorsed. Except item 12 (“fed up or frustrated”), which correlated more highly with role restriction (0.71) than emotional function domain (0.64), all item-total correlations with specified domains were greater than 0.7 (Table 3).

**Table 2.** Item and scale properties of the CHQLQ and HIT-6 at baseline (n = 130).

							Response options^c^	
	Percentage missing	Mean	(SD)	Minimum score	Maximum score	Median	% Floor (minimum score)	% Ceiling (maximum score)
Headache-specific								
CHQLQ^a^								
Items (score range 1–6)								
Role function – Restrictive (RR)								
1. Interfered with family	1.00	3.17	1.26	1	6	3	8.5%	5.4%
2. Interfered with leisure	1.00	3.27	1.20	1	6	3	5.4%	4.6%
3. Difficulty doing work	1.00	3.10	1.12	1	6	3	6.9%	0.8%
4. Getting work done	1.00	3.23	1.08	1	6	3	4.6%	2.3%
5. Limit work concentration	2.00	3.27	1.13	1	6	3	4.6%	0.8%
6. Left too tired	1.00	3.24	1.28	1	6	3	7.7%	3.8%
7. Limited energetic days	1.00	3.46	1.26	1	6	3	3.8%	5.4%
Role function – Prevention (RP)								
8. Had to cancel work	2.00	2.30	1.13	1	6	2	25.4%	1.5%
9. Needed help doing routine tasks	3.00	2.16	1.22	1	6	2	37.7%	1.5%
10. Stop work or daily activities		2.65	1.16	1	6	2	13.8%	1.5%
11. Not able to go to social activities	2.00	2.23	1.19	1	6	2	30.0%	0.8%
Emotional Function (EF)								
12. Often felt fed up or frustrated	0.00	3.88	1.34	1	6	4	2.3%	12.3%
13. Often felt like a burden	0.00	2.72	1.63	1	6	2	33.1%	7.7%
14. Often been afraid of letting others down	0.00	2.95	1.65	1	6	3	23.8%	11.5%
Domain scores (0–100)								
Role restriction (RR) (items 1–7) (n = 124)	3.00	54.21	17.08	17	90	52	0.0%	0.0%
Role prevention (RP) (items 8–11) (n = 124)	4.00	39.01	16.89	17	100	35.5	0.0%	0.8%
Emotional function (EF) (items 12–14) (n = 124)	0.00	52.99	22.84	17	100	50	0.0%	3.8%
								
HIT-6								
Items (score range 1–5)								
1. How often is pain severe	0.00	3.63	0.74	2	5	4	0.00%	10.00%
2. Limit usual daily activities	0.00	3.25	0.85	1	5	3	3.10%	4.60%
3. Lie down	0.00	3.69	1.08	1	5	4	5.40%	24.60%
4. Felt too tired to do work or daily activities	0.00	3.16	0.87	1	5	3	5.40%	3.10%
5. Felt fed up or irritated	0.00	3.62	0.94	1	5	4	1.50%	17.70%
6. Limit ability to concentrate on work	0.00	3.38	0.85	1	5	3	2.30%	7.70%
Index score (0–100)								
HIT-6 (n = 130)^b^	0.00	62.51	6.91	38	78	63	0.00%	1.50%

^a^CHQLQ: Each item has six descriptive response options, ranging from ‘None of the time’ (1 point) to ‘All of the time’ (6 points). Three domain scores: Role function – restrictive (RR); Role function – preventative (RP); and Emotional function (EF) – are calculated as the sum of item responses across each domain, rescaled to a 0–100 scale, where the higher score indicates better headache-related quality of life. A floor effect at item level is where more than 15% of responders score at the minimum (floor) indicating “best” health on the CHQLQ.

^b^HIT-6: Each item has five descriptive response options, with each awarded a specific number of points: “Never” (6 points), “Rarely” (8 points), “Sometimes” (10 points), “Very often” (11 points) and “Always” (13 points). The score is the sum of item (points) responses. The index score ranges from 36 to 78, where scores ≤ 49 indicate little to no impact on life; 50–55 indicates some impact on life; 56–59 indicates substantial impact on life; and ≥ 60 indicates very severe impact on life. A floor effect at item level is where more than 15% of responders score at the minimum (floor) indicating “best” health on the HIT-6.

^c^End effects: Where more than 15% of respondents score the minimum (floor) or maximum (ceiling) score respectively.

**Table 3.** Exploratory (EFA) and confirmatory (CFA) factor analysis: Standardised factor loadings for the proposed three-factor measurement model for the CHQLQ and single-factor measurement model of the HIT-6.

	Structural validity				Internal consistency			
	EFA			CFA	cITC^a^			Cronbach’s alpha
	Eigenvalues >1.0							
Headache-specific	RR	RP	EF		RR	RP	EF	
Proportion variance	0.30	0.20	0.20					
Proportion variance explained	0.43	0.29	0.28					
CHQLQ								
Role function – restrictive (RR)								0.94
1. Interfered with family	**0.59**	0.47		0.80	**0.76**	0.67	0.7	–
2. Interfered with Leisure	**0.71**			0.85	**0.83**	0.72	0.62	–
3. Difficulty doing work	**0.71**			0.89	**0.85**	0.74	0.69	–
4. Getting work done	**0.71**		0.41	0.86	**0.83**	0.72	0.6	–
5. Limit work concentration	**0.63**		0.41	0.78	**0.75**	0.67	0.59	–
6. Left too tired	**0.65**		0.42	0.85	**0.82**	0.75	0.65	–
7. Limited energetic days	**0.71**			0.80	**0.79**	0.65	0.55	–
Role function – preventative (RP)								0.89
8. Had to cancel work	**0.40**		**0.70**	0.83	0.72	0.77	0.58	–
9. Needed help doing routine tasks		0.46	**0.54**	0.78	0.69	0.72	0.65	–
10. Stopped work or daily activities	0.44		**0.64**	0.81	0.71	0.76	0.54	–
11. Not able to go to social activities			**0.65**	0.81	0.7	0.75	0.6	–
Emotional function (EF)								0.87
12. Often felt fed up or frustrated	0.46	**0.48**		0.71	0.71	0.62	**0.64**	–
13. Often felt like a burden		**0.86**		0.93	0.67	0.65	**0.84**	–
14. Often been afraid of letting others down		**0.80**		0.86	0.61	0.57	**0.78**	–
Assessment of model fit:^b^								
Chi-square *p*-value (DF)				<0.001 (74)				
CFI/TLI	0.95			0.95/0.94				
RMSEA (90% confidence interval)	0.079 (0.05, 0.09)			0.086 (0.06, 0.11)				
RMSR	0.03			0.06				
HIT-6 (index score)					–			0.90
1. How often is pain severe				0.71	**0.68**			–
2. Limit usual daily activities				0.85	**0.79**			–
3. Lie down				0.80	**0.75**			–
4. Felt too tired to do work or daily activities				0.85	**0.79**			–
5. Felt fed up or irritated				0.74	**0.72**			–
6. Limit ability to concentrate on work				0.78	**0.75**			–
Assessment of model fit:^b^								
Chi-square (DF)				0.013 (9)				
CFI/TLI				0.974/0.957				
RMSEA (90% confidence interval)				0.101 (0.044, 0.158)				

^a^cITC: Corrected Item-Total Correlations (the extent to which items are adequate reflections of the underlying construct (12,13).

^b^CFA model fit was examined using Comparative Fit Index (CFI), Tucker-Lewis Index (TLI), and the Root Mean Square Error of Approximation (RMSEA).

Note: Values in bold represent corrected item-total correlations between items and their respective total domain scores.

**Table 4.** Two-week test-retest reliability (ICC 2,1), standard error of measurement (SEM) and smallest detectable change (SDC) for the CHQLQ and HIT-6.

		Baseline	Re-test	Change^a^	SEM^b^	SDC individual^c^	SDC group^d^	ICC (95% CI)^e^
	N	Mean (SD)	Mean (SD)	Mean (SD)				
Headache-specific								
CHQLQ (domain scores 0–100)								
RR	67	62.16 (17.05)	67.46 (16.72)	5.30 (11.44)	8.09	22.42	2.74	0.74 (0.55, 0.84)
RP	67	77.04 (18.00)	79.88 (16.99)	2.84 (11.96)	8.459	23.45	2.86	0.76 (0.63, 0.85)
EF	67	63.25 (23.64)	67.04 (24.83)	3.79 (14.96)	10.576	29.32	3.58	0.80 (0.69, 0.87)
HIT-6 (range 35–78)	73	62.56 (7.13)	61.03 (6.77)	−1.53 (3.42)	2.415	6.69	0.78	0.86 (0.75, 0.92)

^a^Self-reported change in headache was captured on a headache-specific health-transition question at 2 weeks.

^b^SEM: Standard Error of Measurement.

^c^SDC_individual_ represents the SDC in individuals and is calculated as: (SEM × 1.96 × √2) (15,16).

^d^SDC_group_ represents the SDC in a group of individuals and is calculated as: (1.96 × √2 × SEM √n, where n is the group size) (6,15,16).

^e^ICC (95% CI): Intra-class correlation coefficient (1,2) with 95% confidence intervals.

There were no missing data for the HIT-6; index scores were computable for all responders. Except for item 1 (pain severity), for which response option 1 (“never”) was not endorsed, all response options were supported. Item-total correlations ranged from 0.68 to 0.79, with five of the six items achieving scores higher than 0.70 (Table 3).

Floor effects (>15%) were identified for three CHQLQ role-prevention items and two emotional function items, suggesting many respondents were not “prevented” from undertaking usual activities or experienced specific emotional difficulties (Table 2). Ceiling effects were observed for two HIT-6 items: >15% respondents indicated they would “always” “lie down” or feel “fed up or irritated” when experiencing a headache, suggesting the importance of these items, but further impact discrimination was impossible.

### Structural validity and internal consistency

Standard loadings and goodness-of-fit indices for the CHQLQ exploratory factor analysis supported the three-factor model, with factor loadings > 0.50 for all items except item 12 (“fed up or frustrated”) (Table 3). Role restriction accounted for the majority (43%) of data total variance. Confirmatory factor analysis produced a good data fit, supporting the CHQLQ’s three-domain model. Confirmatory factor analysis supported the HIT-6 single domain, with all component loadings > 0.70. Cronbach’s alpha ranged 0.87 to 0.94 for the CHQLQ domains and 0.90 for the HIT-6, indicating high internal consistency.

### Reliability

All values for the CHQLQ and HIT-6 exceeded the lower threshold for acceptable test-retest reliability (intra-class correlation coefficient > 0.70), supporting use with groups of patients (Table 4). The standard error of measurement for the CHQLQ domains were 8.09 (role restriction), 8.46 (role prevention) and 10.58 (emotional function), resulting in smallest detectable change for individuals (SDC_individual_) values of 22.42, 23.45 and 29.32, respectively. The corresponding smallest change in scores that can be detected at the group level (SDC_group_) was 2.74 (role restriction), 2.86 (role prevention) and 3.58 (emotional function). This implies that, when using the CHQLQ for individual assessment, changes in people with stable symptoms would need to be greater than 22, 24 or 29 points (between 22% and 29% of total score change) to be distinguishable from measurement error. Alternatively, on a group level, group means would need to differ between 2.74 and 3.58 (up to 4% of total score change) to ensure a true detection of a difference in people with stable symptoms.

The standard error of measurement for the HIT-6 was 2.42, resulting in a SDC_individual_ of 6.69 and SDC_group_ of 0.78. When using the HIT-6 in individual assessment, changes in people with stable symptoms would need to exceed 6.7 points (16% of total score change) to be distinguishable from measurement error. Alternatively, on a group level, group means need to differ by 0.78 (up to 2% of total score change) to be distinguishable from measurement error in people with stable symptoms.

### Construct validity

Most hypothesised associations were supported (Table 5): the CHQLQ’s three domains were strongly associated, with moderate to strong associations with the HIT-6. However, the association between role restriction and the SF-12 mental component score was stronger (moderate) than that observed with emotional function, reflecting the emotional component of the role-restriction domain. (Appendix 3). Similarly, although smaller than hypothesised, associations between role restriction and the HADS were similar or greater than that observed for emotional function, reflecting the limited emotional content of the emotional-function domain specifically, and the CHQLQ generally. Moderate associations between the CHQLQ and the Social Impact Scale and Pain Self-Efficacy Scales reflect the CHQLQ focus on the social impact of headache and pain, respectively.

A strong association with the Pain Self-Efficacy Questionnaire reflects the HIT-6 focus on pain. Apart from the moderate association with the Social Impact Scale, reflecting the HIT-6 emphasis on social impact, small associations with the remaining measures evidence a limited focus on the emotional impact of headache.

### Responsiveness (Table 6)

Of the 105 people completing the 12-week questionnaire, 94 and 100 completed the health-transition question and CHQLQ or HIT-6, respectively.

**Table 5.** Convergent validity matrix between the CHQLQ and comparator measures^a,b^.

	Headache-specific				Generic health status^c^				Domain-specific^d^			
					Profile		Single-item Global	Utility	Emotional Well-being		Pain self-efficacy	Social integration
	CHQLQ domains			Impact	Physical health status	Mental health status		Health Status	Anxiety	Depression		
	RR	RP	EF	HIT-6	SF-12 PCS	SF-12 MCS	EQ-VAS	EQ-5D-5L^e^	HADS-A	HADS-D	PSEQ	SIS-HEIQ
Headache-specific												
CHQLQ												
Role restriction (RR)	1	0.82	0.74	0.73	−0.43	−0.55	−0.48	−0.59	0.33	0.30	−0.68	−0.57
Role prevention (RP)		1	0.69	0.68	−0.46	−0.38	−0.39	−0.59	0.17	0.28	−0.64	−0.50
Emotional function (EF)			1	0.58	−0.22	−0.47	−0.41	−0.46	0.34	0.22	−0.58	−0.43
HIT-6	0.73	0.68	0.58	1	−0.34	−0.35	−0.24	−0.35	−0.18	0.25	−0.61	−0.48

^a^Strength of association (Cohen): small < 0.30; moderate 0.31 to 0.69; strong > 0.70.

^b^All comparator measures detailed in Appendix Table 1.

^c^Generic measures: SF-12: Short-Form 12-item Health Status survey; PCS: Physical Component Score; MCS: Mental Component Score; EQ-VAS: EuroQoL Visual Analogue Scale; EQ-5D-5L: EuroQol 5-dimension Preference-based Utility Index.

^d^Domain-specific measures: Emotional well-being assessed with the HADS: Hospital Anxiety and Depression Scale; A: anxiety scale; D: depression scale; pain self-efficacy assessed with the PSEQ: Pain Self-Efficacy scale; social integration assessed with the SIS – HEIQ: Social Impact Scale of the Health Education Impact Scale.

^e^EQ-5D-5L item content: Stronger focus on physical function (mobility, usual activities, self-care), so stronger association with physical than with emotional domains hypothesised.

**Table 6.** Responsiveness of the CHQLQ and HIT-6 at 12 weeks.

Headache-specific health transition^a^	N	Baseline	3-month	Difference (MIC)^b^	SEM^c^	SDC individual^d^	SDC groupe	ES^f^	SRM^g^
CHQLQ									
Role function – restriction (RR)									
Much better	10	70.50 (12.82)	90.00 (15.58)	19.50 (16.25)	11.49	31.85	10.07	1.521	1.2
Better	19	65.89 (17.31)	76.68 (14.50)	10.79 (10.98)	7.766	21.526	4.94	0.623	0.982
Same	53	62.94 (15.58)	69.98 (13.67)	7.04 (13.35)	9.44	26.167	3.59	0.452	0.527
Worse	12	61.75 (22.76)	58.58 (11.43)	−3.17 (14.35)	10.144	28.117	8.12	−0.139	−0.221
Much worse	0								
Role function – prevention (RP)									
Much better	10	86.80 (10.36)	98.00 (3.89)	11.20 (11.26)	7.964	22.075	6.98	1.081	0.994
Better	19	83.89 (12.41)	89.16 (11.47)	5.26 (7.86)	5.557	15.403	3.53	0.424	0.67
Same	53	78.85 (14.65)	83.26 (13.86)	4.42 (12.71)	8.991	24.921	3.42	0.301	0.347
Worse	12	68.08 (23.18)	67.33 (15.44)	−0.75 (13.61)	9.621	26.667	7.7	−0.032	−0.055
Much worse	0								
Emotional function (EF)									
Much better	10	69.30 (21.80)	88.70 (17.86)	19.40 (21.63)	15.294	42.393	13.41	0.89	0.897
Better	19	68.74 (19.11)	76.74 (17.11)	8.00 (10.78)	7.623	21.13	4.85	0.419	0.742
Same	53	66.32 (21.57)	68.89 (21.55)	2.57 (13.60)	9.618	26.66	3.66	0.119	0.189
Worse	12	58.67 (24.06)	56.42 (24.91)	−2.25 (14.59)	10.318	28.601	8.26	−0.094	−0.154
Much worse	0								
HIT-6									
Much better	11	58.91 (8.31)	51.36 (8.32)	−7.55 (5.18)	3.666	10.16	3.06	−0.908	−1.456
Better	20	62.30 (5.19)	59.15 (4.93)	−3.15 (4.86)	3.436	9.523	2.13	−0.607	−0.648
Same	57	62.44 (6.49)	60.35 (6.59)	−2.09 (5.03)	3.554	9.851	1.3	−0.321	−0.415
Worse	12	64.33 (9.13)	64.75 (7.63)	0.42 (2.43)	1.718	4.761	1.37	0.046	0.172
Much worse	0								

^a^Headache-specific health transition – self-reported change in headache-specific health status at 12-weeks: Much better/better/same/worse/much worse.

^b^MIC: Minimal important change – calculated as the mean change in those who have improved (better/much better) or deteriorated (worse).

^c^SEM: Standard error of measurement.

^d^SDC_individual_ represents the SDC in individuals and is calculated as: (SEM × 1.96 × √2) (15,16).

^e^SDC_group_ represents the SDC in a group of individuals and is calculated as: (1.96 × √2 × SEM √n, where n is the group size) (3,15,16).

^f^ES: Effect size statistic – mean change in scores divided by the standard deviation of the baseline scores.

^g^SRM: Standardised response mean – mean change in scores divided by the standard deviation of the change score.

#### Smallest detectable change (SDC)

The CHQLQ standard error of measurement ranged from 5.60 to 10.31 for participants indicating minimal improvement or deterioration in headache status at 12 weeks. The resultant smallest detectable change for individuals (SDC_individual_) for improvement ranged between 15 (role prevention) to 21 (role restriction), and 26 (role restriction and role prevention) to 28 (emotional function) for deterioration. The corresponding smallest detectable change for groups (SDC_group_) ranged between 3 (role prevention) to 5 (role restriction) for improvement, and 7 (role prevention) to 8 (emotional function) for deterioration. These results imply that when using the CHQLQ for individual assessment, changes of <21 (improvement) or <28 (deterioration) points cannot be distinguished from error. However, much smaller differences are detectable for groups of patients: For groups who indicate minimal improvement, a change from baseline to 12 weeks of >5 points on the role-restriction and emotional-function domains and > 4 on the role-prevention domain are required to demonstrate a change that is greater than measurement error. For groups indicating minimal deterioration, a change of approximately 8 points is required to demonstrate change that is greater than measurement error.

The standard error of measurement for the HIT-6 ranged from 1.7 (deterioration) to 3.5 (improvement). The smallest detectable change at the individual level (SDC_individual)_ was 9.5 and 1.7, and at the group level (SDC_group_) was 2.1 and 1.3 for improvement and deterioration, respectively.

#### Minimal important change (MIC)

Fifty-three of the 94 valid CHQLQ responses at 12 weeks (56%) indicated no change in headache status (mean change in score between 2.57 (SD 13.6) (emotional function) and 7.04 (SD 13.35) (role restriction)). Nineteen reported some (“better”) improvement, with a mean score improvement (minimally important change) of 5.26 (role prevention), 8.00 (emotional function) and 10.79 (role restriction). The remaining 12 participants reported a deterioration (“worse”) in headache status and a mean score deterioration of −0.75 (role prevention), −2.25 (emotional function), and −3.17 (role restriction). The smallest difference between clinically stable and improved participants (i.e. the minimal clinically important difference (MCID)) was 0.84 (role prevention), 3.75 (role restriction) and 5.43 (emotional function).

The minimally important change for the HIT-6 is −3.15 and 0.42 for minimal improvement and deterioration, respectively. The smallest difference between clinically stable and improved patients (minimal clinically important difference) is −1.06 for the HIT-6.

For both measures, the minimal important changes were greater than the smallest detectable change in groups (SDC_group_), indicating that a greater change in score is required to denote “important change” than that required to illustrate change that is greater than measurement error.

#### Criterion-based responsiveness (Figure 1)

**Figure 1.**
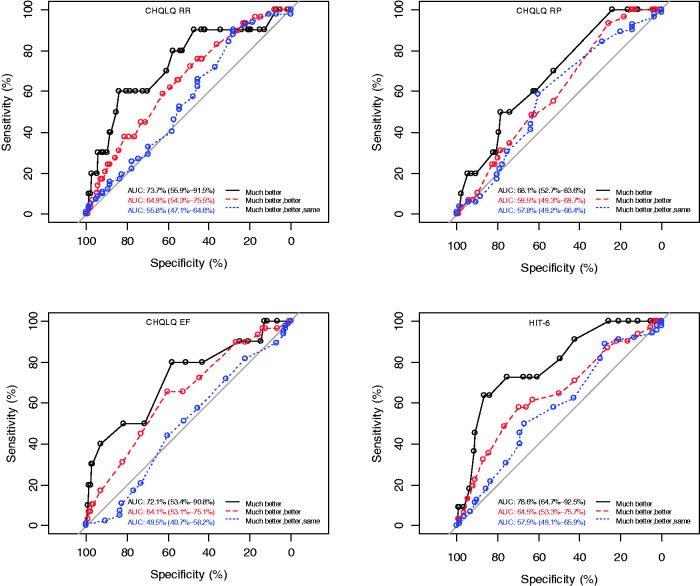
ROC curves. Note: Respondents were dichotomised in three different ways: i) “Much better”: Headache was “much better” versus headache was better, about the same or worse; ii) “much better, better”: Headache was “much better” or “better” (that is, the improved group) versus headache was the same or worsened (the not improved group); and iii) “much better, better, same”: Headache had improved or remained about the same vs. headaches had deteriorated.

Moderate correlations between CHQLQ and HIT-6 change scores with the headache-specific transition item (range −0.35 (emotional function) to −0.45 (role prevention); 0.36 (HIT-6)), supported its use as an external marker of change (24). The higher AUC scores were found when dichotomising patients according to those who were “much better” versus those reporting that they were “better, the same or worse” (Figure 1). Two (role restriction, emotional function) CHQLQ domains exceeded 0.70 (lower bound 95% CI exceeding 0.50), indicating adequate responsiveness. However, the AUC for the role-prevention domain was 0.68, with a lower bound 95% CI of 0.53 (95% CI 0.53–0.84), suggesting limited responsiveness. The AUC for the HIT-6 exceeded 0.70 (95% CI 0.64–0.92). At this level of discrimination, these results suggest adequate responsiveness. However, AUC less than 0.70 were found when participants were grouped differently (Figure 1).

#### Effect size statistics

As hypothesised, both effect size and standardised response means for patient subgroups increased with increased reported improvement on the transition question. Moderate to large effect sizes were found for people reporting some (better) and greater (much better) improvements in headache status at 12 weeks for both the CHQLQ and HIT-6. However, for patients who were unchanged, most values (75%) did not confirm the hypothesis by exceeding 0.2. Small numbers limited interpretation of any headache deterioration.

### Content validity

We interviewed 14 participants (age 21–72 years; nine female) with chronic migraine.

Typically, participants felt the CHQLQ was relevant to their headache experience, specifically welcoming the emotional impact items. However, item overlap – particularly around work – caused participants to refer back to previous items, and increased completion time. Participants described experiencing different headache intensities across the 4-week recall period, requiring judgement as to how they selected the most appropriate response. Double-barrelled items that aligned headache impact on “work” with “leisure activities” or “home” were challenging, as different environments influenced response. Contextual situations – for example, being retired or without dependents – caused participants to rate headache impact differently.

Typically, participants felt that the HIT-6 was relevant, welcoming its brevity and simplicity. However, when considering different headache intensities, the lack of recall period (items 1 to 3) was problematic: A range of recall periods (daily, weekly, fortnightly, monthly, study duration) were reported to assist in completion. The lack of “pain severity” definition (item 1) was problematic – participants made their own judgement of severity before answering. The double-barrelled nature of three items (2, 5, and 6) caused concern. The impact of headache on work, social or household activities could be scored differently – some chose one activity, whereas others “averaged” activities. Ambiguity of meaning was raised for three items: item 3, “wishing” that one could lie down versus “actually” being able to lie down; item 4, what “tiredness” was, and its relationship to headache; and item 5, “fed up or irritated” was perceived as unclear.

## Discussion

This comparative evaluation of the CHQLQ (adapted MSQ v2.1) and HIT-6 found the appropriateness of the CHQLQ as a measure of headache-specific quality of life was supported. Whilst the HIT-6 was similarly strong, concerns over content and relevance were identified.

Although the shortness of the HIT-6 was welcomed, the capture of headache impact was limited when compared to the CHQLQ. The CHQLQ questions addressing the emotional, symptomatic and social impact of headache were appreciated. However, item repetition and redundancy unnecessarily increased completion time. Participants “averaged” responses to manage the CHQLQ’s 4-week recall period; however, the lack of recall period for several HIT-6 items was a greater concern. This limitation was not identified by the quantitative analysis, highlighting the importance of seeking end-user perspectives throughout development and testing. Low levels of missing data supported the acceptability of both measures.

The CHQLQ three-factor model was supported. However, the dual loading of item 12 (“fed-up or frustrated”) on both role-restriction and emotional-function domains suggested multiplicity and interpretation problems (25,28), which was further supported by a stronger item-total correlation with the role-restriction domain than with the emotional-function domain. Qualitative interviews further identified CHQLQ item interplay between domains, describing the importance of context when thinking about headache impact. Similar contextual problems, including a noticeable divide between work and social commitments was described for both the CHQLQ and HIT-6: For example, interviewees reported endeavouring to keep going while at work, but would often cancel social activities.

The magnitude of the between-domain correlations found in our work suggest that the CHQLQ domains are measuring somewhat different aspects of headache-related health and should be retained. Our confirmatory factor analysis and work by Rendas-Baum et al. (26) further support this. High alpha values supported the internal consistency of the three CHQLQ domains. Similarly, high alpha values have been reported for the MSQv2.1 following completion by patients with chronic (27,28) and episodic migraine (8,27).

The single-domain structure of the HIT-6 was supported by both factor analysis and high alpha values, confirming evidence following completion across chronic and episodic headache populations (29,30).

Low reliability was reported for the MSQv2.1 (ICC < 0.70) in patients with “stable” episodic migraine at a 4-week retest (26). Acceptable levels have been reported for the HIT-6 (29,30). The high levels of reliability in this study support application of both measures in groups, with the smallest detectable change (SDC) suggesting a CHQLQ difference in group means greater than 2.74 (role restriction), 2.86 (role prevention), 3.58 (emotional function) and 0.78 for the HIT-6 is required to demonstrate a real change in stable patients.

Associations between different variables provided acceptable evidence of CHQLQ and HIT-6 construct validity, consistent with earlier MSQv2.1 (26,28) and HIT-6 (9,31) evaluations. However, the CHQLQ’s emotional function domain association with alternative measures of emotional wellbeing were less than hypothesised. Given the importance afforded by patients to the emotional impact of headache, the inclusion of measures providing a more nuanced assessment of emotional wellbeing is recommended.

Both measures demonstrated acceptable evidence of responsiveness to headache improvement over 12 weeks. Moreover, two CHQLQ domains (role restriction, emotional function) and the HIT-6 discriminated between dichotomous configurations of self-reported change in health when grouped as “much better” versus “better, same or worse”. The role-prevention domain was unable to discriminate at a higher level of discrimination.

The minimal important change (MIC) values for both measures were greater than the smallest detectable change (SDC) for groups of patients whose headaches had minimally improved, indicating an “important change” for participants is greater than measurement error. The minimally important change values for CHQLQ domains closely approximate those reported following a 3-month completion of the MSQv2.1 by a large US-based, mixed population of migraineurs – role-restriction 5, role-prevention 5 to 7.9, emotional function 8.0 to 10.6 (32).

The HIT-6 minimal important change value closely approximates that determined in US patients with chronic headache (−3.7) (33) and Dutch patients with episodic migraine (−2.5) (34). However, it is smaller than a minimal important change of 8.0 proposed in a Dutch study of patients with tension-type headache (35), where global improvement was defined according to both global improvement *and* a reduction in headache days (greater than 50%). Published minimal important change values for the HIT-6 range from −1.5 (episodic migraine) to −2.3 (chronic daily headache) (7,33–35), approximating the minimal clinical important difference (MCID) of −1.06 found in this study.

This study describes the first, mixed methods comparative evaluation of two generic, headache-related quality of life measures that are not diagnosis specific, in a UK-based cohort of patients living with chronic headaches. Despite the importance of content validity to the relevance and acceptability of measures, few PROM-evaluative studies explore the qualitative aspects of measures (7). While both measures demonstrated comparable psychometric properties, qualitatively the content validity of the CHQLQ was enhanced by the inclusion of items assessing the emotional toll of chronic headache. However, all interviews were conducted with people with definite or probable chronic migraine, potentially limiting the generalisability of these findings to other headache types. While the number of participants were adequate to support a robust evaluation of measurement data quality, reliability and validity, the majority of participants reported “no change” in health at the 12-week follow-up, substantially reducing the numbers available to explore measurement responsiveness. Further evaluations of measurement responsiveness in a larger cohort and following an active intervention will further enhance confidence in the measure’s ability to capture important change, and towards calculation of the minimal important change in score. Evidence suggests that the CHQLQ shows potential for further use in other groups of patients with chronic headache, but this analysis is limited to participants in a feasibility study (for a larger trial) (12). Hence, some caution is required in generalising conclusions and recommendations more widely to the general population of people with chronic headaches.

Since the reported PROM evaluation was explicitly in people without a specific headache diagnosis, the evidence supports application of both measures in trials where recruitment takes place before diagnosis; for example, where diagnosis is part of the intervention, or for epidemiologic surveys – for example, capturing the impact of headache disorders. Further work may be needed to evaluate use of the CHQLQ in other populations of people with chronic headaches where case mix may be different. For example, it might be a useful measure for people with definite chronic migraine and medication overuse headache after further evaluation in that population. That the design of this study did not allow a precise diagnosis for all participants is not a weakness since the evaluation sought to provide evidence in support of the CHQLQ when assessing people with undiagnosed headache disorders.

## Conclusion

This study describes the first comparative evaluation of the new CHQLQ with the HIT-6, demonstrating the added value to be gained from a mixed-methods approach to PROM evaluation. The results of this study, and the consistency with previous evaluations, supports recommendation of the CHQLQ as a high quality, relevant and acceptable measure for chronic headache. In comparison to the HIT-6, for which similarly strong psychometric evidence was reported, the CHQLQ had greater relevance to the wide-ranging impact of chronic headache.

## Clinical implications

- The quality, relevance and acceptability of a new measure of chronic headache quality of life – the Chronic Headache Quality of Life Questionnaire (CHQLQ) – was compared with that of an existing measure, the 6-item Headache Impact Text (HIT-6), following completion in a UK population.
- The CHQLQ better captured the emotional, symptomatic and social impact of chronic headache.
- Both measures had comparable measurement properties.
- The CHQLQ is recommended as a high quality, relevant and acceptable measure for use with patients with chronic headache.

## Appendix

**Appendix:**

**Table 1.** Patient-reported outcome measures collected in the CHESS feasibility trial (all measures were self-completed at baseline, 2 weeks and 12 weeks).

Patient-reported outcome measure				
	Conceptual focus	Response options/recall period	How to score/interpretation	Key reference
Headache-specific				
Chronic Headache Quality of Life Questionnaire (CHQLQ) Modification of the measure involved replacing the term “migraine” with “chronic headache” in the item stem. These changes were supported by the developers (e-mail correspondence: Chris Bell, GSK, January 2016)	Headache-specific quality of life. 14 items assess the functional impact of headaches across three domains: Role restrictions (RR) – restrictions to daily activities caused by headaches Role prevention (RP) (items 8–11) – prevented from engaging in daily activities due to headaches Emotional function (EF) (items 12–14) – the impact of headaches on the individuals emotional well-being	Six descriptive response options, ranging from “none of the time” (1 point) to “all of the time” (6 points) 4-week recall period Trial-specific modification to the MQLQ (v2.1) – the word “migraines” was replaced with “headaches”	Domain item responses are summed: Role restrictions (RR) (items 1–7) Role prevention (RP) (items 8–11) Emotional function (EF) (items 12–14) Domain scores rescaled to a 0–100, where higher scores indicate better headache-related quality of life	Martin. B. C., Pathak D. S., Sharfman. M. I., et al. Validity and reliability of the Migraine-Specific Quality of Life Questionnaire (MSQ Version 2.1). *Headache* 2000; 40: 204–216.
Comparator measures: Headache-specific				
Headache Impact Test (HIT-6).	A six-item measure that purports to provide an overall assessment of headache impact on an individual’s ability to function	Each item has five descriptive response options, with each awarded a specific number of points: “Never” (6 points), “rarely” (8 points), “sometimes” (10 points), “very often” (11 points) and “always” (13 points) No recall period items 1–3 4-week recall period items 4–6	The score is the sum of item (points) responses Index score ranges 36 to 78, with lower scores indicating better health/less impact on life Interpretative guidance: Scores ≤ 49 indicate little to no impact on life; scores 50–55 indicate some impact on life; scores 56–59 indicate substantial impact on life; scores ≥ 60 indicate very severe impact on life	Kosinski, M., et al., A six-item short-form survey for measuring headache impact: the HIT-6. *Qual Life Res* 2003; 12: 963–974
Comparator measures: Generic				
Short Form 12-item Health Survey version 2 (SF-12v2) Website: https://www.optum.com/solutions/life-sciences/answer-research/patient-insights/sf-health-surveys/sf-12v2-health-survey.html	A 12-item, non-preference based measure of generic health status, derived from the SF-36 (Ware 2002). It assesses health across eight domains including physical and social functioning, and mental health	Each item has between three and five descriptive response options 4-week recall period	Item scores are transformed and standardised to compute two summary scales: physical component scale (PSC) and mental component scale (MCS) Scores are based on general population values (range from 0 (substantial limitation) to 100 (no limitation), standardised to have a mean of 50 (SD 10)	Ware J Jr, Kosinski M and Keller SD. A 12-item Short-Form Health Survey: construction of scales and preliminary tests of reliability and validity. *Med* *Care* 1996; 34: 220–233. Jenkinson C, Stewart-Brown S, Petersen S, Paice C. Assessment of the SF-36 version 2 in the United Kingdom. *J Epidemiol Community Health* 1999; 53: 46–50.
EuroQoL EQ-5D-5L Website: https://www.euroqol.org/	Preference-based, generic measure of quality of life Includes five items/domain descriptive system: Mobility, self-care, usual activities, pain and discomfort, anxiety and depression	Five response options per item (no problems/slight problems or moderate problems/severe problems or unable to do (or extreme pain or extreme anxiety/depression) Health status on the day of completion	Simple reporting of item level scores can provide a simple description of health status More usually, responses are used to calculate a utility index score ranging −0.59 to 1.0, where 1.0 is perfect health and a score less than zero is considered a state worse than death (Kind et al. 1997). Utility tariffs generated from a representative sample of the UK adult population (≥ 18 years of age) (Kind, Dolan et al. 1998) were used to derive the utility weights for this study. UK general population normative values for the EQ-5D-3L 0.86 (SD 0.23)	The EuroQol Group. EuroQol: a new facility for the measurement of health-related quality of life. *Health Policy* 1990; 16: 199‒208. Herdman M, Gudex C, Lloyd A, et al. Development and preliminary testing of the new five-level version of EQ-5D (EQ-5D-5L). *Qual Life Res* 2011; 20: 1727–1736.
EuroQoL EQ-Visual Analogue Scale (EQ-VAS)	The second component of the EuroQoL – single items measure of general health	Single 10 cm vertical (thermometer) on which respondents rate their overall health “today”	Overall health today from 0 (worst imaginable) to 100 (best imaginable). UK general population normative values 82.48 (SD 19.96)	
Comparator measures: Domain-specific				
Hospital Anxiety and Depression Scale (HADS)	A 14-item measure of anxiety and depression	Two domains each consist of seven items, with four-point, descriptive response options ranging 0 to 3 Recall period “past week”	Domain item responses are summed: Anxiety: Items 1, 3, 5, 7, 9, 11, 13 Depression: Items 2, 4, 6,8,10, 12, 14 Domain scores range 0 to 21. Interpretative guidance: 0– 7 “normal” 8–10 mild anxiety or depression 11–15 moderate anxiety or depression ≥ 16 severe anxiety or depression	Zigmond, A. S., Snaith, R. P. The Hospital Anxiety and Depression Scale. *Acta Psychiatrica Scandinavica* 1983; 67: 361–370.
Pain Self-Efficacy Questionnaire (PSEQ)	A 10-item measure of an individual’s confidence in performing a particular behaviour or task despite of their pain	Each item has seven possible response options, ranging from “Not at all confident” (0 point) to “completely confident” (6 points) No recall period	Item scores are summed Index score range 0 to 60, where the higher score reflects stronger self-efficacy beliefs	Nicholas, M.K., The pain self-efficacy questionnaire: Taking pain into account. *Eur J Pain* 2007; 11: 153–163.
Social Integration Subscale of the Health Education Impact Questionnaire (heiQ)	A 5-item domain (one of eight) included in the HEiQ. The SIS assesses ability to integrate in society (The HEiQ assesses the impact of patient education programmes in chronic conditions)	Four response options: “Strongly disagree” (1 point), “disagree” (2 points), “agree” (3 points) and “strongly agree” (4 points) No recall period	Item scores are summedIndex score range 5 to 20, where higher scores indicate higher levels of social interaction, a higher sense of support, and confidence in seeking support from others. Lower scores suggest greater feelings of social isolation because of illness	Osborne, R., et al. The Health Education Impact Questionnaire (heiQ): an outcomes and evaluation measure for patient education and self-management interventions for people with chronic conditions. *Patient Educ Couns* 2007; 66: 192–201.
Headache-specific health transition questions	Patient-reported health transition items detailing the size and direction of change in health over a specified period are widely used as patient-derived, external criterion for change. The 2 and 12-week questionnaires included questions that asked patients to rate, overall, if they felt that their headaches were: much better/better/the same/worse/much worse on a 5-point scale			de Vet, C.W., Terwee, C.B., Mokkink, L.B., Knol, D.L. (2011) *Measurement in medicine: A practical guide. Practical guides to biostatistics and epidemiology*. Cambridge University Press.

**Table 2.** Data analysis plan and interpretation

	Description	Analysis and interpretation
Data quality and measurement acceptability		
Completion rates	Item and scale level missing data reported as a reflection of measurement acceptability (6,7,15,16)	Item level and scale level score distribution and the percentage of computable scores reported
Item-total correlation (corrected) (cITC)	The extent to which items are adequate reflections of the common underlying latent construct (15,16)	Corrected item-total correlation. Values between +0.40 and +0.60 suggest moderate levels of inter-item correlation, supporting convergent validity; values greater than +0.70 suggest that there may be item redundancy (15,16)
Interpretability – the ability to assign qualitative meaning to a score or change in score (https://www.cosmin.nl/wp-content/uploads/COSMIN-definitions-domains-measurement-properties.pdf) (14,15)		
End-effects	Where more than 15% of respondents score the minimum (floor) or maximum (ceiling) score (6,15)	–
Minimal important change (MIC)	The MIC is defined as the smallest change in score perceived as important by participants) (14,15)	Calculated as the mean change score for people reporting 'minimal change' in headache at 12 weeks on the headache-specific health transition questionnaire (HTW) (that is, “better” or “worse”)
Structural validity and internal consistency		
Structural validity	Structural validity, a component of construct validity, evaluates measures underlying construction, the presence of sub-domains and item behaviour (6,14–16) Due to the CHQLQ item stem modification, an exploratory factor analysis (EFA) was conducted on baseline data, hypothesising that the original three-factor solution would be retained Confirmatory factor analysis (CFA) was then performed to confirm the proposed three- and one-domain factor structures of the CHQLQ and HIT-6, respectively.	Exploratory factor analysis (EFA) Data was entered into a model with variamax rotation. Absolute item loadings ≥ 0.45 were accepted as sufficient correlation with a principal component to support domain inclusion Confirmatory factor analysis performed using lavaan (http://lavaan.ugent.be/tutorial/tutorial.pdf) in R version 3.3.1 (R Core Team, 2016) by maximum likelihood and full information maximum likelihood (FIML) for the missing data The latent factors were standardised to have mean 0 and standard deviation of 1 to allow free estimation and easily interpretable factor loadings. Factor loadings exceeding 0.3–0.4 were judged to be meaningful (16,17); the model fit was examined using Comparative Fit Index (CFI), Tucker-Lewis Index (TLI), and the Root Mean Square Error of Approximation (RMSEA). A CFI and TLI of > 0.95 and a RMSEA of < 0.05 were considered as adequate fit. For moderate fit, CFI and TLI values of > 0.90 and RMSEA of < 0.08 were used
Internal consistency	Assesses the relationship (interrelatedness) between items within a measure (or sub-domains), reflecting the total number of items and their average correlation (15,16)	The internal consistency of the three CHQLQ domains and the HIT-6 was assessed by calculation of Cronbach’s alpha (15,16) Values between 0.7 and 0.90 suggest a good to excellent agreement between items and the total (domain) score (15,16)
Reliability and measurement error – the degree to which a measure is free from measurement error		
Test-retest reliability	The extent to which scores for patients who have not changed are the same for repeated assessments over time (temporal stability) (6,14–16)	Two-week test-retest reliability was assessed in patients who indicated on health transition item that their headaches had remained stable (15,16) The intra-class correlation coefficient (ICC 2,1) was used to measure the level of agreement between test and re-test (15,16). For group comparisons, levels of reliability greater than 0.70 are recommended, with high levels (> 0.90) required for individual-level assessment
Measurement error Standard Error of Measurement (SEM) Smallest Detectable Change (SDC)	The systematic and random error of a patient’s score that is not attribute to true changes in the construct to be measured (https://www.cosmin.nl/wp-content/uploads/COSMIN-definitions-domains-measurement-properties.pdf) The extent of absolute measurement error is determined by calculation of the Standard Error of Measurement (SEM) The SEM supports score interpretation by accounting for variability, or error, in measurement - only a change greater than measurement error is considered “real” (15,17) The Smallest Detectable Change (SDC) represents the smallest change in score that is greater than measurement error The SDC allows one to rule out measurement error (i.e. distinguishing measurement error from true change) when assessing the reliability of a self-reported measure to detect change in health status Thus, a score change greater than the SDC value is necessary to provide evidence of true change (improvement or deterioration) in health-status	Standard Error of Measurement (SEM) Calculated using a two-way random effects model (6,15) The SEM was subsequently converted into the smallest detectable change (SDC). The SDC was calculated both for individuals and groups (6,15,19) SDCindividual = SEM × 1.96 × √2 SDCgroup = 1.96 × √2 × SEM √n (where n is the group size)
Construct and content validity – the degree to which a measure measures what it purports to measure		
Content validity – qualitative evidence in support of purported measurement focus	The degree to which the content of the PROM measures the construct(s) it purports to measure (https://www.cosmin.nl/wp-content/uploads/COSMIN-definitions-domains-measurement-properties.pdf 14-16 Evidence that details the clarity of measurement content in terms of relevance, comprehensiveness and comprehensibility with respect to the purported measurement focus (construct of interest) and the target population (for example, chronic headache) (14–16)	Semi-structured cognitive interviews were conducted with a purposive sample of patients with confirmed chronic headache to explore the relevance, acceptability, clarity and comprehensiveness of the measures, as per the four stages of cognitive processing: (21,22) - Comprehension: The process of making sense of the questions and developing a response - Memory retrieval: The process of accessing relevant information to enable a response - Judgement: The process to determine if memory retrieval is accurate and complete - Response mapping: The process by which an appropriate response is selected Several overarching questions sought to explore how patients determined an improvement in their headache, and if specific questions were missing. Interviews were continued until thematic saturation was achieved Participants were interviewed within 24 h of questionnaire self-completion. Verbal prompting was used to facilitate the interview process. To counteract fatigue bias, the CHQLQ and HIT-6 were alternately completed All interviews were audio recorded, transcribed verbatim, and checked for accuracy (VN) Framework analysis (23) and cross-case comparison was used to generate themes, informed by PROM item content, relevance and the additional over-arching questions NVivo software was used to organise the data Data was independently explored by two researchers (VN, KH). Emergent themes were discussed and interpreted with a third researcher (FG)
Construct validity – quantitative evidence in support	The degree to which PROM scores are consistent with hypotheses, and based on the assumption that the PROM is a valid measure of the construct to be measured (https://www.cosmin.nl/wp-content/uploads/COSMIN-definitions-domains-measurement-properties.pdf) 14-16 Assessed by correlating the scores for separate measures to assess the convergent validity of related domains (Pearson’s correlation coefficient): it was expected that related constructs would correlate more strongly	Hypothesised theoretical associations between the three domains of the CHQLQ and comparator measures were considered a priori (Appendix Table 3). The RF and RP domains of the CHQLQ and HIT-6 measure related domains and hence a stronger association than with the EF domain was hypothesised. Similarly, the SF-12 PCS and EQ-5D-5L have a greater focus on physical aspects of health, and a stronger association with the RL and RP domains was hypothesised than with the EF domain; but a stronger association between the EF and the SF-12 MCS was hypothesised. A stronger association between the EF domain and the HADS-A and HADS-D was hypothesised. Several items within all three domains of the CHQLQ consider the social impact of headache, and hence moderate to strong association with the HEiQ SIS was hypothesised. The focus of the PSEQ is one’s ability (and confidence) in managing pain and engaging in (largely) physical and social activities; hence a moderate association with the RL and RP domains, but small association with the EF domain was hypothesised
Responsiveness – the ability of a measure to detect real change in health over time that is greater than measurement error		
Smallest detectable change (SDC) in score	To understand the smallest change in score that is greater than measurement error in patients reporting change in headache at 12 weeks	Standard error of measurement (SEM) and smallest detectable change (SDC): To represent the smallest change in score that is greater than measurement error in those patients reporting change in headache in the headache-specific health transition question at 12 weeks, we calculated: - The absolute measurement error at 12 weeks (SEM)- The SEM was subsequently converted into the smallest detectable change (SDC) (14–16,19,20) - The SDC was calculated for both individuals and groups: - SDCindividual = SEM × 1.96 × √2 - SDCgroup = 1.96 × √2 × SEM √n (where n is the group size) Minimal important change (MIC): - Calculated as the mean change in those who report a minimal improvement/deterioration on headache-specific health transition question at 12 weeks Minimal important difference (MID): - Calculated as the mean change in score of those who were “somewhat better” minus the mean change in those who were the same on headache-specific health transition question at 12 weeks
Criterion-based assessment of responsiveness	To understand the ability of measures to discriminate between patients whose headache had improved or deteriorated. This change was captured by the patient-reported headache-specific health transition question (an external criterion or “gold standard” of change) (6,14–16)	External criterion: First, the level of correlation between change scores on CHQLQ and HIT-6 and the transition item was calculated; a level of agreement of 0.3 to 0.5 was considered acceptable as a marker of change (24) ROC analysis and area under the curve (AUC) Receiver operating characteristic (ROC) curves were used to assess the ability of the measures to discriminate between patients who had improved or deteriorated, as per patient-self report of change in headache status at 12 weeks Respondents were dichotomised in three ways: i) Headache “much better” versus headache “better, about the same or worse” ii) Headache “much better or better” (that is, the improved group) versus headache “the same or worse” (the not improved group) iii) Headache had :improved or remained about the same” versus headaches had “deteriorated” The larger the area under the curve (AUC) (on a scale of 0.5 (no discriminatory power) to 1.0 (perfect discrimination)), the more sensitive the measure at detecting differences in the external indicator An AUC of > 0.70 is considered as sufficiently discriminatory (6,15,16), whilst an AUC of 0.5 suggests no discriminatory power
Effect size (ES) and Standardised response mean (SRM) statistics	Distribution-based assessment (longitudinal validity): a priori defined hypotheses about the expected magnitude of differences in changes between defined groups (defined by responses to the 12-week headache-specific transition item) were proposed and tested (15) Effect size classification (15): Small 0.20 Moderate 0.50 Large 0.80	Effect size (ES): Mean change divided by baseline SD Standardised response mean (SRM): Mean change divided by SD of the change score Both values were calculated for sub-groups of patients in each health transition category. Given that this was a feasibility study with patients not receiving any intervention and the follow-up period was short, the main hypotheses to be tested for responsiveness were: • ES and SRM would be < 0.2 for patients who reported no change in headache • ES and SRM would be > 0.2 for patients reporting slight improvement • ES and SRM would be > 0.5 for patients reporting improvement (much better) • ES and SRM would be greater for patients indicating an improvement in their headache than those indicating no change

**Table 3.** Convergent validity matrix: Hypothesized associations (size and direction) between CHQLQ and comparator measures.

	Headache-specific		Generic				Domain-specific			
			Profile		Single item – general	Utility	Emotional well-being			
	Headache-specific QL	Impact	Physical function	Mental well-being		Health status	Anxiety	Depression	Pain self-efficacy	Social integration
CHQLQ	CHQLQ	HIT-6	SF-12 PCS	SF-12 MCS	EQ-VAS	EQ-5D-5L	HADS - A	HAS - D	PSEQ	SIS-HEiQ
Role Restriction (7 items)	Strong, positive with RP; Moderate positive with EF	Strong, positive	Moderate to strong, negative	Small to moderate, negative	Moderate, positive	Moderate* (to strong), positive	Small to moderate, positive	Small to moderate, positive	Moderate, positive	Moderate, positive
Role Prevention (4 items)	Strong, positive with RR; Moderate positive with EF	Strong, positive	Moderate to strong, negative	Small to moderate, negative	Moderate, positive	Moderate* (to strong), positive	Small to moderate, positive	Small to moderate, positive	Moderate, positive	Moderate, positive
Emotional Function (3 items)	Moderate, positive with RR and RP	Moderate to strong, positive	Small, negative	Moderate to strong, negative	Small to moderate, positive	Moderate, positive	Moderate to strong, positive	Moderate to strong, positive	Moderate, positive	Moderate, positive

Note: Strength of association (Cohen): Small < 0.30; moderate 0.31 to 0.69; strong > 0.70.

*EQ-D item content: Stronger focus on physical function (mobility, usual activities, self-care), so stronger association with physical than with emotional domains hypothesised.
